# Impact of livestock industry on climate change: Case Study in South Korea — A review

**DOI:** 10.5713/ab.23.0256

**Published:** 2023-11-01

**Authors:** Sun Jin Hur, Jae Min Kim, Dong Gyun Yim, Yohan Yoon, Sang Suk Lee, Cheorun Jo

**Affiliations:** 1 Department of Animal Science and Technology, Chung-Ang University, Anseong 17546, Korea; 2 Farm and Table Co. Ltd., Seoul 06339, Korea; 3 Department of Agricultural Biotechnology, Center for Food and Bioconvergence, and Research Institute of Agriculture and Life Science, Seoul National University, Seoul 08826, Korea; 4 Department of Food and Nutrition, Sookmyung Women’s University, Seoul 04310, Korea; 5 Department of Animal Science and Technology, Sunchon National University, Suncheon 57922, Korea

**Keywords:** Carbon Footprint, Climate Change, Environment, Greenhouse Gases, Livestock Industry

## Abstract

In recent years, there has been a growing argument attributing the primary cause of global climate change to livestock industry, which has led to the perception that the livestock industry is synonymous with greenhouse gas (GHG) emissions. However, a closer examination of the global GHG emission by sector reveals that the energy sector is responsible for the majority, accounting for 76.2% of the total, while agriculture contributes 11.9%. According to data from the Food and Agriculture Organization of the United Nations (FAO), the total GHG emissions associate with the livestock supply chain amount to 14.5%. Within this, emissions from direct sources, such as enteric fermentation and livestock manure treatment, which are not part of the front and rear industries, represent only 7%. Although it is true that the increase in meat consumption driven by global population growth and rising incomes, has contributed to higher methane (CH_4_) emissions resulting from enteric fermentation in ruminant animals, categorizing the livestock industry as the primary source of GHG emissions oversimplifies a complex issue and disregards objective data. Therefore, it may be a misleading to solely focus on the livestock sector without addressing the significant emissions from the energy sector, which is the largest contributor to GHG emissions. The top priority should be the objective and accurate measurement of GHG emissions, followed by the development and implementation of suitable reduction policies for each industrial sector with significant GHG emissions contributions.

## INTRODUCTION

Until now, it has been believed that the cause of global warming was the extensive use of fossil fuels since the Industrial Revolution. In the 2020s, however, a claim was made that the livestock industry was affecting global warming, and was the main cause of the climate crisis. This claim has gained strength in recent years, leading to the perception that the livestock industry causes greenhouse gas (GHG) emissions [1]. In fact, the education sector, which is in the public domain, is expanding its use of vegetarian meals, and central and local governments are recommending a vegetarian diet or adopting various programs to replace livestock products with other types of food. The argument that the livestock industry is a main cause of the climate crisis originated from the “Livestock’s Long Shadow”, which is the published report of the Food and Agriculture Organization of the United Nations (FAO) [2]. At the time, they estimated that the total GHG emissions of the livestock supply chain were 18%, and reported that the livestock sector emits more GHGs than the transportation sector worldwide [2]. However, livestock is an important source of micronutrients and proteins for mankind, providing about 17% of total energy and 33% of proteins. Based on population increase, urbanization, and income growth, the demand for livestock products has contributed to job and income generation for 1.3 billion people and accounted for about 33% of total agricultural production [3]. As such, it is a major industry that produces livestock products, which contributes significantly to human lives. Nevertheless, the majority of reports are on climate change, rather than this, which shows that many journalists perceive that the livestock industry accelerates the climate crisis. However, in general, the livestock industry does not perceive itself as being the main cause of climate change, in contrast to what green groups and the media tend to put forward. This is because, although the livestock industry is emitting GHGs, the amount is relatively lower than that of other sectors, such as the energy sector. Based on available scientific evidence and objective logic, this paper aims to point out the errors in various arguments that have identified the livestock industry as the main cause of GHG emissions, and suggest rational response strategies for climate change.

## WORLDWIDE GREENHOUSE GAS EMISSION STATUS

GHG emissions are typically categorized into total and net emissions. Total emissions encompass emissions from all sectors, excluding the Land-Use, Land Use Change, and Forestry (LULUCF) sector, while net emissions include emissions from the LULUCF sector [5]. On a global scale, the energy sector accounts for a substantial 76.2% of total GHG emissions, with agriculture contributing 11.9%. A closer examination of the energy sector reveals that electricity/heat generation constitutes the largest share at 31.9%, followed by transportation (16.9%), manufacturing and construction (12.6%), fugitive emissions (5.9%), buildings (5.9%) and other fuel combustion (3.0%) (Table 1).

**Figure 1:**
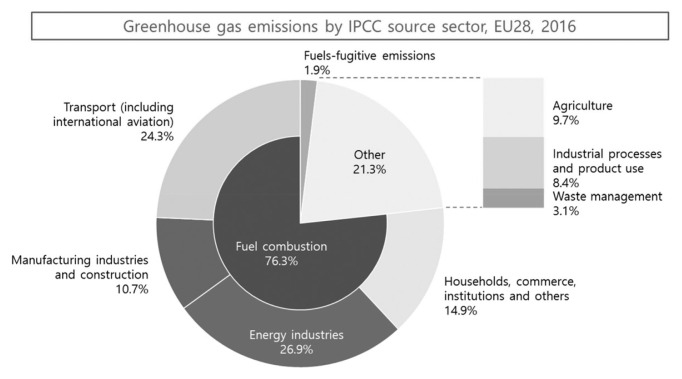
Greenhouse gas emissions by European Industrial Sector [6].

In terms of greenhouse gas emissions by industrial sector in Europe, the energy industries had the highest rate at 26.9%, the transport sector accounted for 24.3%, and the agriculture sector accounted for 9.7% of the total (Figure 1). In line with the 2014 IPCC report [7], GHG emissions by industry sectors reveal that the electric energy sector holds the most significant share at 25%, followed by the agriculture and forestry sector (24%), and the industrial sector (21%). According to data from the Food and Agriculture Organization (FAO), the livestock sector is responsible for emitting 7.1 GtCO_2_eq annually, constituting 14.5% of all human-induced emissions [2]. Analyzing net GHG emissions by major regions shows that China is the largest emitter, followed by the United States, the European Union, India, and Russia. Brazil ranks sixth in terms of carbon emissions worldwide; however, its net emissions are lower than those of Japan, a country with limited landmass and no significant forests (Table 2).

**Figure 2:**
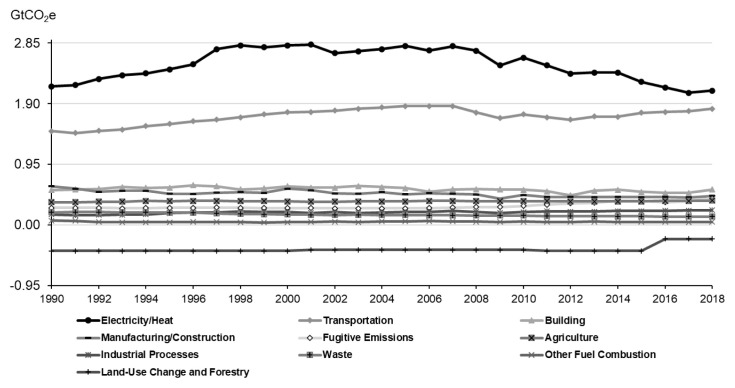
Greenhouse gas emissions by sector in the United States [10].

In the United States, the overwhelming majority of GHG emissions, 91%, are attributed to energy sector. Within the energy sector in the United States, the most significant contributor is the electricity/heat generation process, accounting for 36.3% of total GHG emissions. Approximately 62% of electricity generation in the United States relies on burning fossil fuels, predominantly coal and natural gas. In 2018, the transportation sector accounted for 30.4% of GHG emissions, primarily arising from the operation of vehicles such as cars, trucks, ships, trains, and airplanes powered by fossil fuels. In the agriculture sector, GHG emissions constituted 6.6%, originating from livestock, particularly cattle, as well as emissions associated with soil and rice production. Conversely, the land-use and forestry sector serve as a crucial GHG sink, absorbing 229 million tons of GHGs (Figure 2).

## GREENHOUSE GAS EMISSION STATUS IN SOUTH KOREA

**Figure 3:**
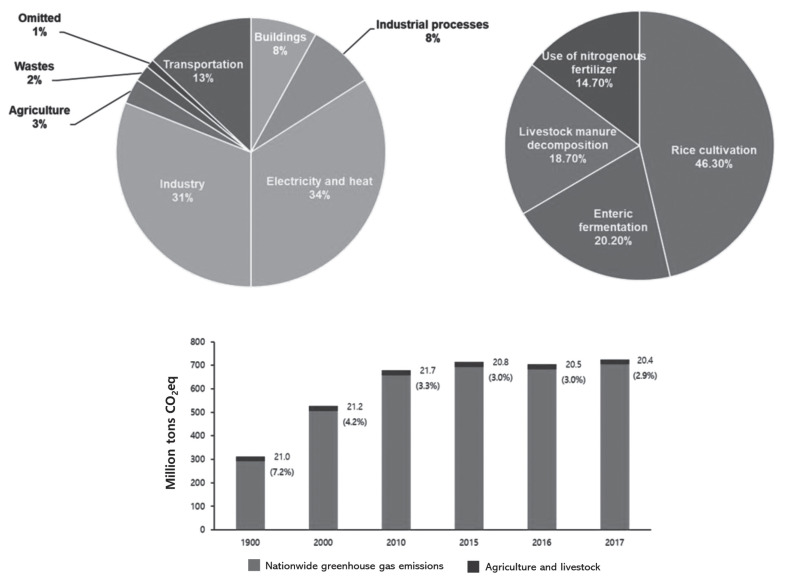
Greenhouse gas emissions by industrial sector in South Korea [8,12,13].

In 2018, South Korea's total GHG emissions reached 727.6 million tons CO_2_eq, representing a significant increase of 149.0% compared to the 292.2 million tons CO_2_eq reported in 1990. This also marked a 2.5% increase from the previous year (2017), when total emissions amounted to 709.7 million tons CO_2_eq (Table 3) [11]. When examining the contribution of each sector to these emissions, the energy sector shares at 86.9%, making it the largest contributor. It is followed by industrial processes at 7.8%, agriculture at 2.9%, and wastes at 2.3%. Notably, since the 1990s, GHG emissions have increased by a 149%. Within this period, emissions in the energy sector increased by 163.1%, the industrial process sector by 178.7%, and the waste sector by 64.7%. In contrast, the agriculture sector witnessed only a marginal 1% increase (Table 3; Figure 3).

Specifically, within the livestock sector, GHG emissions in 2013 amounted to 9.9 million tons CO_2_eq, constituting 1.4% of the nation's total GHG emissions. This accounted for 47.6% of all emissions in the agriculture sector. Breakdowns reveal that emissions from enteric fermentation were 4.4 million tons CO_2_eq, making up 91.7% of the total enteric fermentation emissions. Among these, dairy cattle contributed 23.9%, while Korean native cattle (Hanwoo) and beef cattle accounted for 67.8%. Emissions from livestock manure treatment totaled 5.5 million tons CO_2_eq, with Hanwoo and beef cattle leading the charge at 36.6%. They were followed by swine at 29.4%, poultry at 19.1%, and dairy cattle at 12.5% [14].

It's important to note that the Ministry of Environment's estimations indicated that 9.6 million tons of GHGs were generated by 3,202,000 Hanwoo/beef cattle in 2019. This equated to 2.99 tons of GHG per head. However, further analysis demonstrated that in 2020, this figure reduced to 2.95 tons of GHGs per head, with a total emission of 9.9 million tons from 3,353,000 head of cattle [15]. This raised objections from the livestock industry, which argued that these figures were excessive and, from a mathematical standpoint, implausible.

Greenhouse gas emissions from the livestock industry account for 1.29% of total emissions. When comparing global greenhouse gas emissions by country in 1990, they are in that order: China, the United States, India, Russia, and Japan. In 2018, Korea's greenhouse gas emissions were 727.6 million tons, ranking 11th in the world. The proportion is estimated at 1.51% [8]. Looking at South Korea's share of emissions by greenhouse gas, CO_2_ accounts for 91.4%, CH_4_ 3.8%, N_2_O 2.0%, HFCs 1.3%, SF_6_ 1.2%, and PFCs 0.4% (Table 4). Comparing by major greenhouse gases, CO_2_ increased by 163.8% compared to 1990 and 2.2% compared to 2017 (Table 5). Compared to 1990, CH_4_ emissions decreased by about 8.4%, and N_2_O increased by 62.9% (Table 5).

Recent data from the Rural Development Administration (RDA) provides more accurate insights. It reveals that CO_2_ emissions from enteric fermentation amounted to 315 kg per head due to shortened fattening periods, while CO_2_ emissions in the livestock manure treatment process accounted for 150 kg per head. When examining the CH_4_ emission factor for each livestock type to calculate CH_4_ emissions per head, the following was observed: dairy cattle emitted 118 kg annually through enteric fermentation and 36 kg in the livestock manure treatment process; Hanwoo/beef cattle emitted 47 kg through enteric fermentation and 1 kg in the livestock manure treatment process; and swine emitted 1.5 kg through enteric fermentation and 3 kg in the livestock manure treatment process. These findings suggest that the figures presented by the Ministry of Environment [8] were overestimated by more than 4 times compared to the RDA data (The Ministry of Environment estimated 2.99 tons GHG/head/yr, while the RDA estimated around 600 kg GHG/head/yr).

## DOES THE LIVESTOCK SECTOR CONSTITUTE 51% OF WORLDWIDE GREENHOUSE GAS EMISSIONS?

In 2009, the Worldwatch Institute [17], a private environmental research institute, released a report claiming that the livestock sector was responsible for 51% of total GHG emissions. This assertion contended that numerous factors were omitted from the GHG estimates provided by the FAO. It is important to emphasize that these arguments, as presented in this report, are widely considered unscientific and have not gained recognition from academia or relevant international organizations, including the IPCC and FAO. Indeed, data from a 2013 FAO report [18] estimated that the total GHG emissions attributable to the livestock supply chain amounted to 14.5%. Of this, emissions from the direct emission sectors, namely enteric fermentation and livestock manure treatment, which do not encompass the front or rear industry sectors, accounted for 49%.

In the livestock industry, this comparison involved assessing the GHG production across the entire supply chain, from the cultivation of feed crops to feed manufacturing and transportation, livestock breeding, transportation, slaughtering, processing, sales, and disposal. Conversely, the transportation sector's comparison was based on the sum of GHG emissions when transportation vehicles, such as cars, ships, airplanes, and trains, were operational. To ensure a fair comparison, emissions in the transportation sector should encompass the full life cycle, including vehicle manufacturing, operation, disposal, and the production, processing, and distribution of petroleum-type fuels. When strictly considering direct emissions, the transportation sector and the livestock industry contributed 16.9% and 7% of global emissions, respectively. In South Korea, transportation accounted for 13.5%, while livestock constituted only 1.3%. On a global scale, the livestock sector's GHG emissions are less than half of those generated by the transportation sector, and in South Korea, merely one-tenth of the emissions produced by transportation (Table 6).

**Figure 4:**
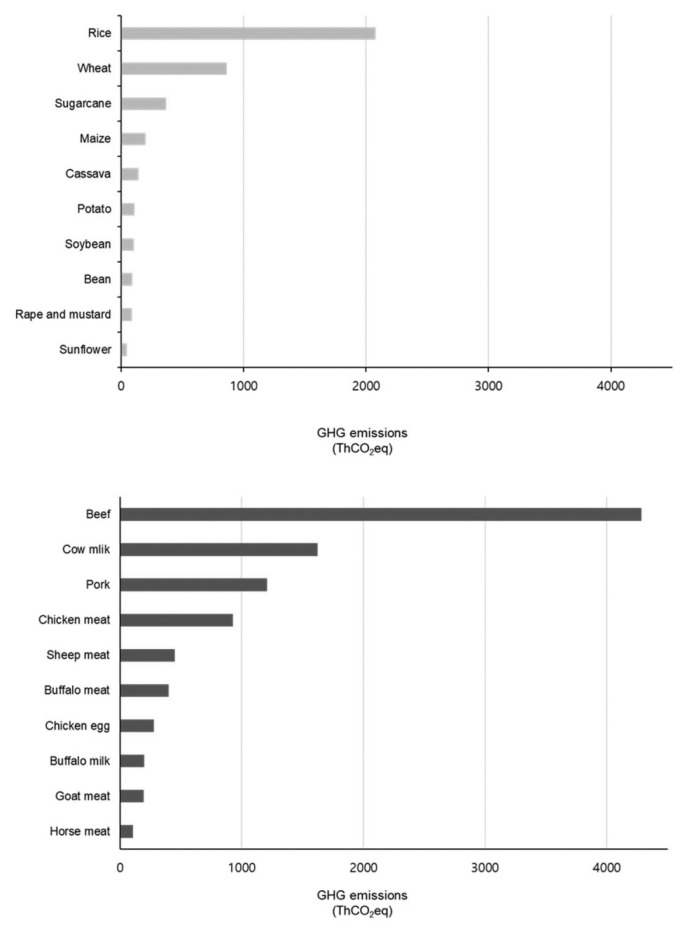
Greenhouse gas emissions by agricultural and livestock product [20].

According to Euro-CASE [6], rice farming has been a significant contributor to CH_4_ emissions during the 20th century, necessitating emission control measures. Jeong et al [11] projected a decrease in total CH_4_ emissions from rice cultivation, estimating 6.271 million tons CO_2_eq in 2021 to decrease to 6.122 million tons CO_2_eq in 2025 and 6.051 million tons CO_2_eq in 2030. This considerable CH_4_ release stems from the decomposition of organic materials, particularly fertilizer, during rice cultivation. Consequently, even if vegetarian diets were to replace livestock products, the impact on GHG emissions would be relatively limited. Research by Lee et al [19] indicated that producing 1 kg of rice results in the emission of 1.40 kg of CO_2_, with CH_4_-induced carbon emissions from rice cultivation representing a substantial 71.1%. N_2_O emissions from nitrogen fertilization constitute 11.8%, while carbon emissions during composite fertilizer manufacturing account for 7.6%. Surprisingly as shown from the following data, beef is a major GHG emissions, and contrary to what is commonly believed, rice cultivation also emits about half of the GHG emissions of beef (Figure 4).

Data from Table 7 reveals that crop-based CO_2_ emissions attributed to energy consumption in the agriculture sector are 24.4% for vegetables, the highest share, followed by 23.1% for Hanwoo/beef cattle and 15.1% for rice. In regions where rice is a dietary staple, expanding vegetarian diets as a strategy to reduce GHG emissions is expected to yield limited results. Given that calories are primarily derived from rice in such regions, a reduction in livestock product consumption would likely necessitate increased rice consumption to meet calorie requirements. In 2018, the agriculture sector's emissions constituted 2.9% of the total national emissions, amounting to 21.2 million tons CO_2_eq in total, representing a 1.0% increase from 1990. Emissions were distributed across sectors, with the rice cultivation sector accounting for 29.7%, followed by agricultural lands/soil (25.8%), livestock manure (23.1%), and enteric fermentation (21.2%). Notably, GHG emissions from livestock manure treatment reached 4.9 million tons in 2018, marking a 5.9% increase from 2017.

## ESTIMATION OF GREENHOUSE GAS EMISSIONS IN LIVESTOCK PROCESSING AND DISTRIBUTION PROCESSES

The RDA in South Korea recently released findings from a study titled "Research on the Development of Carbon-Reducing Livestock Product Distribution Technology in Response to Global Warming." This study focused on calculating the carbon emissions associated with the production and distribution of major livestock products, including cattle and swine. For 1 kg of beef, the study revealed that 2.1 g of CO_2_ were emitted during a 10-day aging process, 24.3 g during 26-day storage, and 308 g during a 3-day displaying period (Table 8) [22]. These emissions are approximately three times higher than those generated during the aging, storage, and displaying of 1 kg of pork. This difference can be attributed to the longer aging and storage periods required for beef compared to pork.

Further analysis of carbon emissions in the production, slaughtering, processing, and distribution stages of Hanwoo beef and pork revealed that 16.55 kg of CO_2_ are produced per 1 kg of Hanwoo meat. In the case of Hanwoo, emissions reach 17.58 kg during slaughtering, 27.41 kg in processing, and 27.75 kg in distribution. Comparing these figures with those of pork, it was evident that Hanwoo beef emits approximately 7 to 8 times more CO_2_ in the production and slaughtering stages and about 2.5 times more in the processing and distribution stages.

Additionally, a report on carbon emissions related to the distribution of domestic and imported beef indicated that Hanwoo beef results in 27.75 kg of CO_2_ emissions per 1 kg of beef (Table 9), whereas imported beef from the U.S. generates 92 kg of CO_2_ per 1 kg. In this sense, the distribution of the imported beef is associated with emissions approximately three times higher than that of Hanwoo beef.

**Figure 5:**
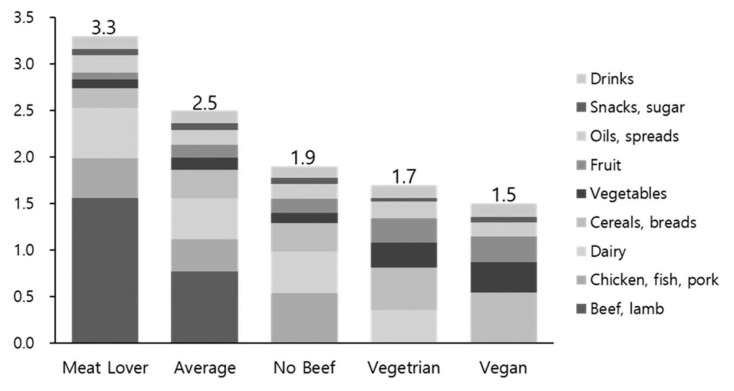
Greenhouse gas emissions by diet type [21]. All estimates are based on average food production emissions for the U.S. Footprints include emissions from supply chain losses, consumer waste, and consumption. Each of the four example diets is based on 2,600 kcal of food consumed per day, which in the US equates to around 3,900 kcal of supplied food.

In terms of per capita CO_2_ emissions based on dietary choices according to USDA data, individuals with high meat consumption release 3.3 kg of GHGs per day, while the average emissions per person are 2.5 kg. Conversely, those abstaining from beef have emissions of 1.9 kg, similar to the 1.7 kg emitted by vegetarians. Vegans exhibit the lowest emissions at 1.5 kg per person, contributing to GHG reductions of about 50% compared to individuals with high meat consumption (Figure 5).

## CONTROVERSY SURROUNDING GREENHOUSE GAS EMISSIONS FROM THE LIVESTOCK INDUSTRY

On November 29, 2006, the UN FAO issued a statement titled "Livestock a Major Threat to Environment" [23]. The statement opens with a comparison of GHG emissions between cattle farming and automobile usage, referencing "Livestock's Long Shadow," a report published by the FAO [2]. According to this report, the livestock sector contributes 18% of GHG emissions, surpassing those from the transportation sector. It highlights the livestock sector as a significant driver of soil and water quality degradation. The report argues that livestock are among the primary contributors to today's most pressing environmental issues, demanding immediate attention and action.

The analysis within the report illustrates that in the process of raising livestock and utilizing land for this purpose, the livestock sector is responsible for 9% of total CO_2_ emissions resulting from human activities, a staggering 65% of human-related N_2_O emissions (which possesses a Global Warming Potential [GWP] 296 times that of CO_2_), and 37% of human-induced CH_4_ emissions (23 times the GWP of CO_2_). The report indicated on the unsustainable development of the livestock sector, which has led to a range of environmental problems worldwide, including water pollution in Europe, uncontrolled deforestation in South America, desertification in Africa and Mongolia, heightened global greenhouse effects, reduced biodiversity, and the diversion of crops that could be used for human consumption to feed animals, exacerbating food shortages. The report attributes these adverse effects to the actions of corporate livestock farms in the U.S. and South America, as well as nomadic livestock herders, who have prioritized short-term gains over long-term sustainability. Nevertheless, it's important to note that the report also provides sector-specific countermeasures and mitigation strategies to facilitate the transition toward a "sustainable livestock industry." Many countries have institutionalized these methods, and even before the report's publication, environmental regulations had been adopted to reduce environmental impacts.

Following the FAO's release of a summary of "Livestock's Long Shadow" via a press release, there has been a surge in citations that predominantly emphasize the negative environmental aspects of livestock farming. This trend has sometimes led to misconceptions, as local livestock-related issues are erroneously perceived as global challenges.

### Unfair comparison

The GHG emissions estimations provided by the FAO are the result of a comprehensive assessment that involves estimating and aggregating GHG production across various industries within the value chain associated with the livestock sector. This estimation for life cycle assessment of GHG emissions from the livestock sector encompasses a wide spectrum of activities, including land-use changes for cultivating feed crops, feed crop cultivation itself, the transportation of feed crops, the manufacture of compound feeds, the transportation of compound feeds, livestock rearing, manure treatment, livestock transport (for milk and eggs), slaughtering, dairy processing, egg collection and treatment, livestock product storage (refrigeration and freezing), transportation of livestock products, and their subsequent sale.

In contrast, GHG emissions in the transportation sector are calculated differently. These emissions are typically determined by multiplying the GHG emissions per kilometer traveled by the number of vehicles and the average driving distance in the case of automobiles. Given the variance in GHG emissions based on vehicle types and fuel sources, these factors are considered in the calculations. Moreover, emissions from ships, trains, and airplanes are also factored in to arrive at the overall GHG emissions for the transportation sector.

The claim that livestock emissions exceed those of all vehicles, trains, ships, and airplanes combined arises from this uneven comparison. However, when comparing only the CH_4_ emissions during the enteric fermentation process and the CH_4_ and N_2_O emissions during livestock manure treatment, which are presented as emissions from the livestock sector, it becomes evident that emissions from the transportation sector are substantially higher. Equations comparing GHG emissions between cattle and vehicles show that a single unit of vehicles emits GHGs equivalent to 4.2 Hanwoo cattle or 1.6 dairy cattle.

**Figure 6:**
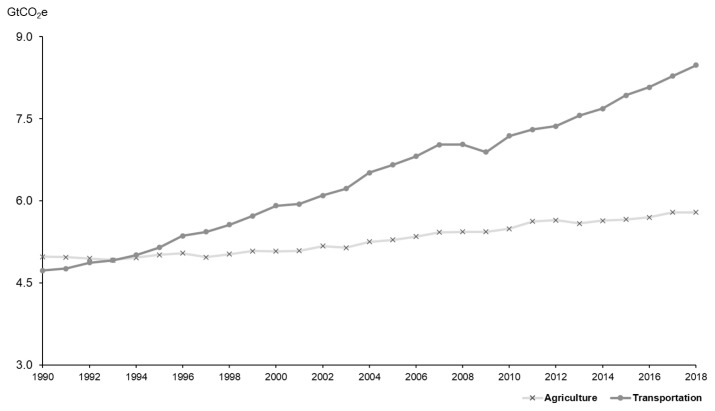
Comparison of GHG emissions between the agriculture and transportation sectors [10].

Figure 6 and Table 10 provide a comparison of GHGs between the agriculture and transportation sectors over the years. While vehicle emissions were less than those from agriculture until 1995, they increased significantly afterward, driven by a surge in the number of vehicles and enhanced international cooperation. In 2018, emissions were 5,817.7 million tons in the agriculture sector and 8,257.7 million tons in the transportation sector, accounting for 12% and 17% of total emissions, respectively. Consequently, the claim that the livestock sector emits more GHGs than the transportation sector, a belief that gained traction following the publication of the FAO's "Livestock's Long Shadow" report [2], is disputed.

**Figure 7:**
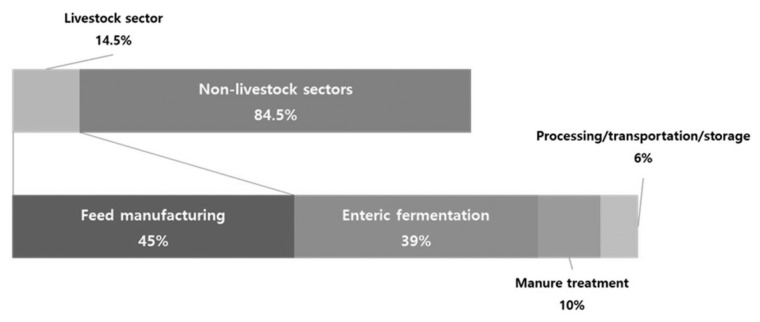
Worldwide greenhouse gas emissions in the livestock supply network [18].

After the FAO estimated and reported GHG emissions across the entire livestock supply network in "Livestock's Long Shadow," it became evident that the total GHG emissions in the supply network—not just those from "enteric fermentation" and "livestock manure treatment", the direct emission sectors of livestock—should be considered when analyzing GHG data. The "Tackling Climate Change through Livestock" report published by the FAO [18] similarly emphasized that GHG emissions in the livestock supply network totaled 7.1 Gt, contributing to 14.5% of global GHG emissions (Figure 7). It categorized the major GHG emission sources within the livestock sector into four distinct categories, as previously mentioned in this article: feed production, enteric fermentation, manure treatment, and other processing and transportation, accounting for 45%, 39%, 10%, and 6% of GHG emissions, respectively [18]. While this comprehensive estimation method provides a holistic view of the livestock industry's impact on climate change, it has led to the perception that the livestock sector emits excessive GHGs.

This method of estimating GHG emissions, which includes emissions from the front and rear industry sectors of livestock such as feed crop cultivation and feed manufacturing, should be reconsidered, especially given the perception of excessive GHG emissions by the livestock industry. Reducing GHG emissions in the front and rear industry sectors should be a responsibility of each respective industry and its workers, rather than being solely attributed to livestock farms.

## ISSUES IN THE CALCULATION OF CARBON DIOXIDE EMISSION FROM LIVESTOCK RESPIRATION

**Figure 8:**
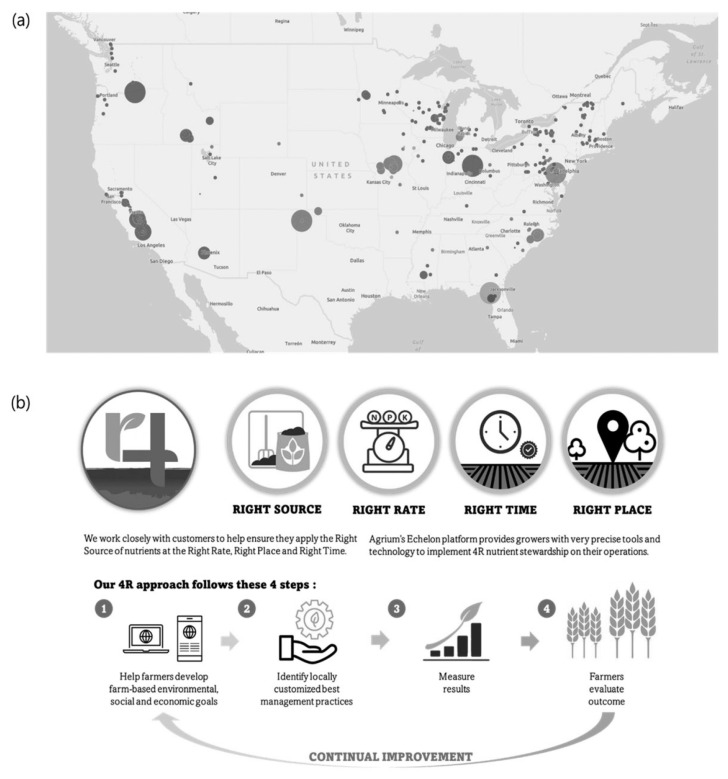
Carbon reduction programs of the livestock sector in North America. (a) US EPA, AgStar infographics [24]; (b) concept and implementation procedures of 4R Nutrient Stewardship [25].

CO_2_, CH_4_, and N_2_O are considered typical GHGs but are not classified as such unless they are emitted through human activities. CO_2_ emitted by wild animals or humans during respiration, CH_4_ released during the enteric fermentation process in wild cows and buffalos, CO_2_, CH_4_, and N_2_O generated during the decomposition of wildlife manure or natural vegetation like leaves and grass, and CH_4_ produced by lakes and wetlands can indeed contribute to significant greenhouse effects. However, these gases are not classified as GHGs since they are considered part of natural carbon and nitrogen cycles in the environment and are not perceived to cause global warming (Figure 8).

On the contrary, all gases resulting from human-made activities are classified as GHGs. Goodland and Anhang [17] reported that CO_2_ exhaled by livestock during respiration should be classified as GHG emissions. However, the Kyoto Protocol assumes that CO_2_ released by livestock while consuming feeds is in a net-zero state because it is reabsorbed during the photosynthesis process of plants [2].

Goodland and Anhang's claim that the livestock sector accounts for 51% of GHG emissions implies that the remaining sectors are responsible for the other 49%. This figure encompasses all GHG emissions from energy production, transportation, industries and industrial processes, and buildings. However, it is challenging to reconcile with common sense that GHGs emitted from energy production, transportation, and various industrial processes for a global population of 7 billion are less than those emitted from the livestock sector. Figures published by other organizations are considerably lower, making it difficult to accept Goodland and Anhang's assertion that the livestock sector disproportionately contributes to GHG emissions. Table 11 displays global GHG emissions reported by various organizations. While discrepancies exist in the figures due to variations in measurement periods and criteria, GHG emissions from the livestock sector are much lower than those claimed by Goodland and Anhang.

GRID-Arendal estimated that the combined emissions from the livestock and feed sectors accounted for 5.1% of total GHG emissions [26], and the World Resources Institute (WRI) estimated that GHG emissions from livestock and feeds constituted 5.8% of the total [4]. EDGR approximated that the entire agriculture sector, encompassing livestock and related industries, contributed to 11.6% of total GHG emissions. UNCC estimated that the agriculture sector, including livestock and associated industries, contributed to 8.6% of GHG emissions. IEA estimated that the total agricultural production, including livestock and feed production, was responsible for 11.9%. Furthermore, FAO estimated that annual GHG emissions from livestock and feeds (CO_2_eq; GHG emissions converted into the equivalent amount of CO_2_) stood at 7,516 million tons, constituting 11.8% of the total [29]. These estimates indicate that the argument made by Goodland and Anhang regarding GHG emissions from the livestock sector lacks consensus within the academic community.

## STRATEGIES FOR GREENHOUSE GAS REDUCTION IN THE LIVESTOCK SECTOR

### The need establish net-zero strategy based on the national situation

GHG emissions across various sectors vary significantly depending on the region and country. Factors such as the level of industrial development, climate conditions (e.g., cold or warm regions), and a country's overall economic development all influence the GHG emissions profile of each sector. Table 12 underscores that the energy sector is the primary source of emissions, prompting international efforts to transition from fossil fuels to renewable energy sources. South Korea's carbon neutrality program primarily revolves around energy transition because, in 2018, a staggering 86.9% of the nation's total emissions stemmed from the energy sector. The core of energy sector measures lies in transitioning to renewable energy sources that do not produce GHGs, supported by technological advancements to enhance energy efficiency.

In contrast, for countries like Brazil, a significant focus lies on reducing emissions from the agriculture sector, as it constitutes the largest share of total emissions among all sectors. However, the issue arises when the same pressure to reduce GHG emissions in the livestock sector, which is pertinent to certain countries like Brazil, is uniformly applied to countries where the livestock sector plays a minor role. The extensive media coverage of climate change related to the livestock industry serves as evidence that people perceive the South Korean livestock sector as a substantial GHG emitter. Brazil, as the world's largest beef cattle raising country, indeed exhibits significant GHG emissions within the agriculture sector, where the livestock sector constitutes a substantial proportion. Conversely, South Korea's agriculture sector has a relatively minor role in terms of GHG emissions. In regions where livestock plays a dominant role, such as Brazil, India, the U.S., and Europe, the livestock sector accounts for a relatively large share of agricultural emissions. However, in East Asian countries like South Korea and Japan, where rice is the staple food, rice cultivation emerges as a more substantial contributor to GHG emissions than livestock. Therefore, in East Asian countries like South Korea, focusing on the livestock industry alone is unlikely to yield significant GHG reduction effects. In South Korea, the livestock sector contributes to about 1.4% of total domestic emissions, whereas the energy sector accounts for a substantial 86.9%. Hence, achieving net-zero emissions in South Korea primarily hinges on energy transition.

### GHG reduction effects of altering the slaughter age for Hanwoo cattle

From 2010 to 2016, approximately 2.51 million Hanwoo steers were slaughtered. Among them, nearly 47% were aged between 30 and 32 months, and around 20% were aged below 29 months. With gradual improvements in specification management and feed technology in South Korea, the 28-month specification program for Hanwoo cattle, supported by domestic and foreign feed companies, has gained traction. If the rearing period is shortened by 3 months, CH_4_ emissions per head of Hanwoo cattle can be reduced by approximately 10.4% (equivalent to about 465 kg CO_2_eq) based on the shortened rearing duration. With a 28-month rearing period, CH_4_ emissions per head amount to 124 kg, representing a reduction of 15 kg (or 5.05 kg per month) compared to a 31-month rearing period. When applied to steers in South Korean, this reduction equates to approximately 182,000 tons of CO_2_eq annually, signifying a potential 3.7% reduction in GHG emissions from Hanwoo cattle.

### GHG reduction methods for feed plants

A study conducted by the Korea Rural Economic Institute (KREI), commissioned by the National Agricultural Cooperative Federation (NACF) of South Korea, aimed to enhance the productivity of 21 mixed feed plants. According to the KREI's report, productivity gains of 10% to 15% could be achieved if NACF-affiliated mixed feed plants specialized in small/medium or large livestock [30]. This increased productivity arises from the fact that each mixed feed plant typically operates a single production facility, resulting in frequent operational pauses for cleaning when producing multiple product types. The research project conducted by KREI found that by specializing each plant in small/medium or large livestock, productivity could increase by up to 15%, leading to annual feed cost savings of approximately 22.6 to 27.6 billion KRW. Consequently, productivity enhancements in mixed feed plants can also translate into GHG reduction.

### Widespread adoption of low-methane feed technology

South Korea employs the Emission Trading System (ETS) as a prominent carbon reduction program. Under the ETS, major GHG-emitting companies are allocated GHG emission allowances, allowing those emitting less than their allocated quotas to sell their excess allowances to companies exceeding their limits, thereby generating profits. Presently, significant attention is drawn to the development and supply of low-CH_4_ feeds aimed at reducing GHG emissions from the enteric fermentation process in ruminants, such as cows. Emissions from cattle represent the most substantial portion of GHG emissions in the livestock sector, making the efficacy of GHG reduction in this sector contingent upon the extent to which low-CH_4_ feeds can mitigate CH_4_ emissions.

The challenge lies in attributing GHG emissions reductions from the development and supply of low-CH_4_ feeds. These reductions are presently credited to livestock farms, representing a front-end industry. However, this perspective overlooks the role of feed industry development and supply in reducing overall GHG emissions. Therefore, it's imperative to allocate some of the GHG emissions reductions from the use of low-CH_4_ feeds to the feed industry as a means to incentivize emissions reduction.

## CONCLUSION

The perception that the livestock industry is the primary driver of climate change has been from unfair comparisons, unscientific GHG estimation, and misunderstandings related to GHG estimation methods within the livestock supply chain. To rectify this misconception, there is an urgent need for efforts to counter misinformation, necessitating collaborative actions between government bodies and industry stakeholders. It is crucial to develop and promote sustainable livestock practices as part of the broader endeavor to transition towards a lower-carbon society. Furthermore, reliance on inaccurate statistical data can divert attention and resources from industries that contribute significantly more GHGs than the livestock sector. Consequently, efforts solely within the livestock industry may prove insufficient in addressing overall GHG reduction goals. Thus, it becomes imperative to prevent the dissemination of erroneous information, and the livestock sector should engage proactively in initiatives aimed at GHG emissions reduction. This approach should also include a thorough analysis of the reasons behind the livestock sector's disproportionate association with GHG emissions.

## Figures and Tables

**Table 1:** Greenhouse gas emission (million tons CO_2_eq) share of each industrial sector worldwide

Emission sector	Emissions	Emission share (%)
Total excluding land-use change and forestry	47,552.14	97.2
Total including land-use change and forestry	48,939.71	100.0
Energy (76.2%)		
Electricity/heat	15,590.95	31.9
Transportation	8,257.73	16.9
Manufacturing/construction	6,158.32	12.6
Fugitive emissions	2,883.39	5.9
Building	2,882.53	5.9
Other fuel combustion	1,452.02	3.0
Industrial processes	2,902.68	5.9
Agriculture	5,817.65	11.9
Land-use change and forestry	1,387.56	2.8
Waste	1,606.86	3.3

World Resources Institute [4].

**Table 2:** Total greenhouse gas emission (million tons CO_2_eq) status by country

Rank	Country	1990	2010	2017	2018	1990–2017 (Increase/decrease, %)	2016–2017 (Increase/decrease, %)
1	China	-	10,543	12,476	-	-	2
2	USA	6437	6,982	6,488	6,677	1	−1
3	India	-	2,137	2,793	-	-	5
4	Russia	3,188	2,058	2,155	2,220	−32	3
5	Japan	1,270	1,303	1,289	1,238	2	−1
6	Brazil	550	917	968	-	76	2
7	Iran	-	810	922	-	-	2
8	Indonesia	267	682	899	-	237	9
9	Germany	1,249	942	894	858	−28	−2
10	Canada	603	691	714	729	18	1
11	Republic of Korea	292	656	710	728	143	2%
12	Mexico	455	669	705	-	59	−0.04
13	Saudi Arabia	165	525	630	-	281	1
14	Australia	425	541	557	558	31	1
15	South Africa	347	539	545	-	57	1

Ministry of Environment Korea [8], United Nations Framework Convention on Climate Change [9].

**Table 3:** Greenhouse gas emissions (million ton CO_2_eq) by year in South Korea

Sector	1990	2000	2010	2017	2018	Increase/decrease (%) over 1990	Increase/decrease (%) over 2017
Total emissions (excluding LULUCF)	292.2	502.9	656.3	709.7	727.6 (100%)	149.0	2.5
Net emissions (including LULUCF)	254.4	444.5	602.5	668.3	686.4 (94.3%)	169.8	2.7
Energy	240.4	411.8	566.1	615.7	632.4 (86.9%)	163.1	2.7
Industrial processes	20.4	50.9	53.0	55.9	57.0 (7.8%)	178.7	1.9
Agriculture	21.0	21.4	22.1	21.0	21.2 (2.9%)	1.0	1.1
LULUCF	−37.8	−58.4	−53.8	−41.5	−41.3 (−5.7%)	9.3	−0.5
Wastes	10.4	18.8	15.2	17.2	17.1 (2.3%)	64.7	−0.7

Ministry of Environment Korea [8].

**Table 4:** Emission of greenhouse gas (million tons CO_2_eq) in South Korea

Greenhouse gas	CO_2_	CH_4_	N_2_O	HFCs	PFCs	SF_6_	Total emissions
Emissions in 2018	664.7	27.7	14.4	9.3	3.2	8.4	727.6
Emission share (%)	91.4	3.8	2.0	1.3	4.0	1.2	100

Ministry of Environment Korea [8].

**Table 5:** Changes in emissions (million tons CO_2_eq) of each greenhouse gas in South Korea

Greenhouse gas		1990	1995	2000	2010	2016	2017	2018	Increase/Decrease (%)	

									1990	2017
CO_2_	Emissions	252.0	384.2	443.7	595.3	637.4	650.2	664.7	163.8	2.2
	Share (%)	86.2	88.6	88.2	90.7	91.9	91.6	91.4		
CH_4_	Emissions	30.2	28.8	27.8	27.6	27.0	27.4	27.7	−8.4	1.0
	Share (%)	10.3	6.6	5.5	4.2	3.9	3.8	3.8		
N_2_O	Emissions	8.8	14.2	17.9	13.0	13.5	13.9	14.4	62.9	3.5
	Share (%)	3.0	3.3	3.6	2.0	1.9	2.0	2.0		

Greenhouse Gas Inventory and Research Center [16].

**Table 6:** Comparison of greenhouse gas emissions (million tones CO_2_eq) between agriculture and transportation sectors

Sector	Worldwide emissions		Emissions in South Korea	
	
	Emissions	Share in total emissions (%)	Emissions	Share in total emissions (%)
Total emissions	48,939.7	-	727.6	-
Agriculture (Livestock)	5,817.7	11.9 (7.0)	21.2 (9.4)	2.9 (1.3)
Transportation	8,257.7	16.9	98.1	13.5

Ministry of Environment Korea [8], Climatewatch [10].

**Table 7:** CO_2_ emissions by crop and livestock based on energy consumption (tons) in the agriculture sector in South Korea

Category		CO_2_	

		Emissions	Share (%)
1	Rice	341,720	15.1
2	Barley	47,888	2.1
3	Wheat	2,735	0.1
4	Other grains	14,420	0.6
5	Vegetables	551,694	24.4
6	Fruit	121,164	5.4
7	Beans	9,973	0.4
8	Potatoes	9,733	0.4
9	Oilseed crops	1,185	0.1
10	Medicinal crops	27,487	1.2
11	Other edible crops	5,538	0.2
12	Fiber crops	66	0.0
13	Leaf tobacco	16,728	0.7
14	Ornamental plants	172,277	7.6
15	Natural rubber	0	0.0
16	Seeds and seedling	4,751	0.2
17	Other non-edible crops	26	0.0
	Total crops	1,327,385	58.7
18	Dairy	229,920	10.2
19	Hanwoo/beef cattle	521,202	23.1
20	Pigs	124,498	5.5
21	Poultry	36,654	1.6
22	Other livestock	19,857	0.9
	Total livestock	932,131	41.3
	Total agriculture	2,259,516	100.0

Wilson [21].

**Table 8:** Estimated greenhouse gas (GHG) emissions (g CO_2_eq) in the distribution stage in South Korea

Functional unit	GHG emissions
1 kg of beef	2.1
Aging (10 days on average)	
Storage (26 days on average)	24.3
Displaying (3 days on average)	308
1 kg of pork	
Aging (6 days on average)	0.9
Storage (6 days on average)	5.4
Displaying (3 days on average)	132

Choi et al [5].

**Table 9:** Carbon emissions (kg CO_2_eq) in production, slaughtering, processing, and distribution stages of Hanwoo and pork

Category	Production	Slaughtering	Processing	Distribution
Hanwoo	16.55 (1 kg of raw meat from a 30 month old)	17.58 (1 kg of pre-rigor)	27.41 (1 kg of refrigerated beef)	27.75 (1 kg of refrigerated beef)
Pork	2.62 (1 kg of raw meat from standard hog)	2.47 (1 kg of pre-rigor)	12.31 (1 kg of refrigerated pork)	12.44 (1 kg of refrigerated pork)

Choi et al [5].

**Table 10:** Comparison of GHG emissions (million ton CO_2_eq) between the agriculture and transportation sectors

Year	1990	1995	2000	2005	2010	2015	2017	2018
Agriculture	4,997.8	5,038.2	5,094.1	5,307.6	5,515.2	5,691.6	5,821.1	5,817.7
Transportation	4,609.0	5,024.9	5,770.3	6,498.6	7,011.9	7,717.0	8,078.5	8,257.7

Climatewatch [10].

**Table 11:** Greenhouse gas emissions from livestock and relevant industries

Country	Organization	GHG emissions		Source

		Emission source	Emissions (%)	
Norway	UNEP/GRID-Arendal	Livestock, feed	5.1	[26]
		Agricultural lands	6.0	
		Use of energy for agriculture	1.4	
		Other emissions from agriculture	0.9	
Europe	Emission Database for Global Atmospheric Research (EDGAR)	Total agricultural production (including livestock and relevant industries)	11.6	[27]
	United Nations Farmwork Convention on Climate Change (UNCCC)	Total agricultural production (including livestock and relevant industries)	8.6	[9]
World	World Resources Institute (WRI)	Livestock, feed	5.8	[4]
		Agricultural lands	4.1	
		Logging	2.2	
	International Energy Agency (IEA)	Total agricultural production (including livestock and relevant industries)	11.9	[28]

**Table 12:** Omitted or underestimated greenhouse gas emissions (million tons CO_2_eq) in the livestock sector

Sector	U.S.	EU	Brazil	South Korea	China	Japan
Agriculture	385.25	389.55	496.1	14.18	672.87	21.56
Building	550.68	435.04	20.38	51.09	542.13	106.11
Bunker fuels	144.85	259.59	16.74	46.67	63.82	36.25
Electricity/heat	2,103.71	1,108.45	88.44	373.7	5,214.2	561.86
Energy	5,271.21	2,902.77	437.33	617.23	10,318.51	1,090.42
Fugitive emissions	301.58	55	8.68	5.21	693.66	2.3
Industrial processes	233.91	166.07	28.99	77.85	1,166.29	67.97
Manufacturing/construction	458.79	−233.92	387.94	−45.8	−649.43	−32.05
Other fuel combustion	94.75	378.83	86.98	71.97	2,667.43	191.68
Transportation	1,762.23	118.29	41.18	13.6	284.08	23.92
Waste	133.24	807.16	191.66	101.66	917.02	204.56
Land-use change and forestry	−229.27	108.7	70.22	9.62	197.57	6.8

Climatewatch [10].
